# Retinal Organoid Technology: Where Are We Now?

**DOI:** 10.3390/ijms221910244

**Published:** 2021-09-23

**Authors:** Zuming Zhang, Zihui Xu, Fa Yuan, Kangxin Jin, Mengqing Xiang

**Affiliations:** 1 State Key Laboratory of Ophthalmology, Zhongshan Ophthalmic Center, Sun Yat-sen University, Guangzhou 510060, China; gnimzhz101@163.com (Z.Z.); xzh46@163.com (Z.X.); yuanf28@mail2.sysu.edu.cn (F.Y.); 2 Guangdong Provincial Key Laboratory of Brain Function and Disease, Zhongshan School of Medicine, Sun Yat-sen University, Guangzhou 510080, China

**Keywords:** retinal development, retinal organoid, disease models, inner cell layer degeneration, transplantation, stem cells

## Abstract

It is difficult to regenerate mammalian retinal cells once the adult retina is damaged, and current clinical approaches to retinal damages are very limited. The introduction of the retinal organoid technique empowers researchers to study the molecular mechanisms controlling retinal development, explore the pathogenesis of retinal diseases, develop novel treatment options, and pursue cell/tissue transplantation under a certain genetic background. Here, we revisit the historical background of retinal organoid technology, categorize current methods of organoid induction, and outline the obstacles and potential solutions to next-generation retinal organoids. Meanwhile, we recapitulate recent research progress in cell/tissue transplantation to treat retinal diseases, and discuss the pros and cons of transplanting single-cell suspension versus retinal organoid sheet for cell therapies.

## 1. Introduction

Many human retinal diseases such as Retinitis Pigmentosa (RP), Age-Related Macular Degeneration (AMD), and glaucoma cause irreversible retinal degeneration and damage. Evolutionarily lower-ranked vertebrates such as amphioxus and zebrafish possess a powerful retinal regenerative capacity. In comparison, retinas in birds have very limited regenerative ability, while mammalian retinas are almost impossible to regenerate once damaged [1]. Although with mouse and other model organisms, researchers have gained an important understanding of the mechanisms of retinal development and disease occurrence, the structure and cell composition of the human retina are quite different from those of model organisms such as mice, which hinders the application of laboratory research to clinical practice. The emergence of retinal organoid technology brought high hope in the field [2]. Retinal organoids derived from Human Embryonic Stem Cells (hESCs) or Human-Induced Pluripotent Stem Cells (hiPSCs) can simulate the development of human retina in vitro, potentially provide personalized treatment based on a patient’s genetic background, and can be used for drug screening, gene therapy, and cell transplantation.

It is convenient to obtain hiPSCs to induce human retinal organoids. The unlimited sources and few ethical issues make the retinal organoid a popular tool for studying the pathogenesis of retinal diseases and graft treatments. Although considerable progress has been made recently, there are still many problems that cannot be ignored, such as the variations among batches of organoids, the methods to obtain cells suitable for transplantation from retinal organoids [3,4], the safety and long-term survival of transplanted cells or tissues in vivo [5], and the efficiency of neural circuit formation in the host. This review aims to introduce the historical background of the production of retinal organoids, summarize current methods to induce retinal organoids and to establish disease models, and address the existing problems of retinal organoid technology and possible solutions. We also discuss the research progress of the transplantation of single-cell suspension and lamellar tissues from retinal organoids.

## 2. The Pre-Organoid Era of Inducing Retinal Cells from Progenitors or Stem Cells

### 2.1. Inducing Retinal Cells by Transplantation or Co-Culture

**Figure 1:**
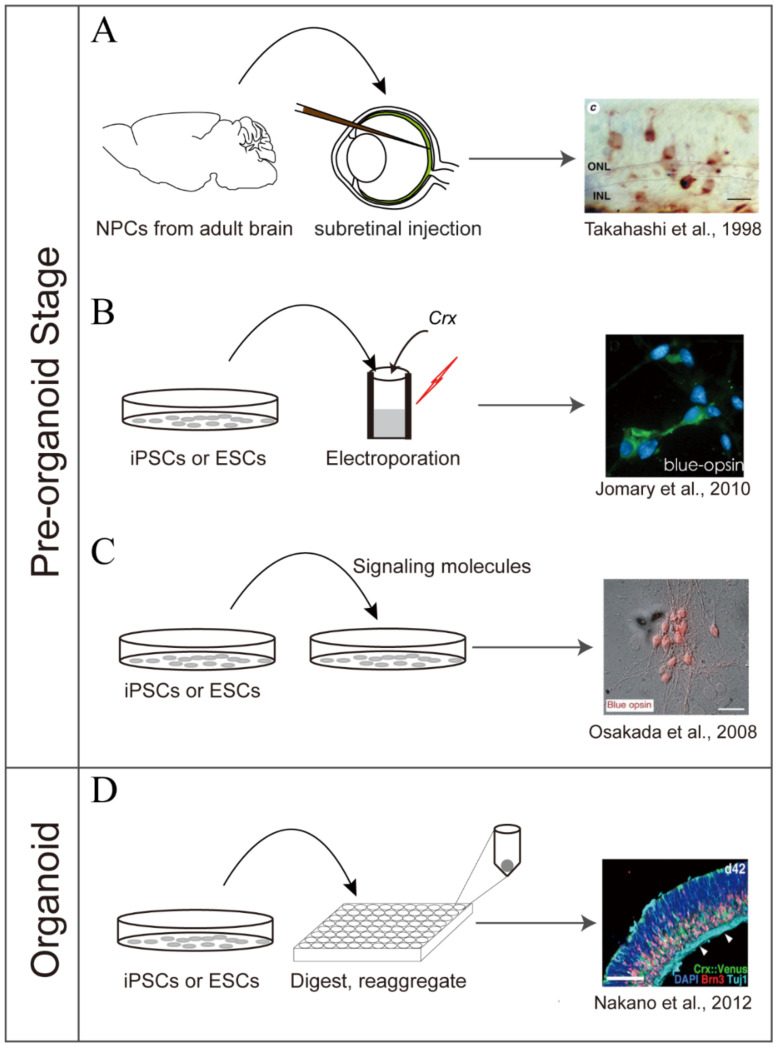
The methodological history of inducing retinal cells. (**A**–**C**) During the pre-organoid stage, the retinal cells were induced by transplanting NPCs/RPCs into the retina in vivo, misexpressing retinal transcription factors in the ESCs/iPSCs, or culturing these cells with proper signaling molecules. (**A**) NPCs purified from the adult rat hippocampus were injected intravitreally and they differentiated into photoreceptor-, amacrine-, bipolar-, horizontal-, and Müller-like cells in the host retina. (**B**) The cone-rod homeobox gene *Crx* was overexpressed in mouse retinal stem cells or human ocular stem cells and induced them to the cone-like photoreceptor. (**C**) The ESCs and iPSCs can also be induced into photoreceptors by signaling molecules, such as SHH, DKK1, retinoic acid, and taurine. (**D**) The retinal organoids with laminar structures were induced from mouse/human ESCs and were capable of recapitulating the characteristics of native retinas. DKK1, Dickkopf WNT Signaling Pathway Inhibitor 1; ESCs, embryonic stem cells; INL, inner nuclear layer; iPSCs, induced pluripotent stem cells; NPCs, neural progenitor cells; ONL, outer nuclear layer; SHH, sonic hedgehog. The right four panels are adapted with permission from Refs. 2, 9, 13, and 21, respectively.

Before the retinal organoid technology was introduced, researchers found that when Retinal Progenitor Cells (RPCs) or other neural stem cells were transplanted into retinas [6,7,8,9], or co-cultured with retinal cells or explants [10,11], they were able to differentiate into retinal cells such as photoreceptors, amacrines, or Retinal Ganglion Cells (RGCs) (Figure 1A). Apart from the well-known tumorigenic and immunogenic risks of transplanting stem cells, there are many common unresolved issues entangled with the transplantation. Few of the transplanted cells could survive, and even fewer could functionally integrate into the neural circuits. The integrated cells differentiated randomly, usually to amacrine-like and rod-like cells. The co-culture of RPCs or stem cells with retinal cells has an inherent disadvantage because it is difficult to distinguish between the cells differentiated from progenitor/stem cells and the pre-added retinal cells.

### 2.2. Inducing Retinal Cells by Over-/Mis-Expressing Retina-Related Transcription Factors or Culturing with Signaling Molecules

In order to obtain cells from a more stable source, researchers focused on self-renewable retinal stem cells and RPCs, which could readily differentiate into retinal cells upon neuronal induction. It was found that overexpressing *Crx* (Cone-Rod Homeobox) in mouse or human retinal stem cells could direct them to differentiate into photoreceptors [12,13], and transplantation of which could partially restore the vision of *Pde6b*^rd1^ mice [14]. Alternatively, ESCs and iPSCs provide nearly unlimited cell sources for inducing retinal cells. Misexpression of *Pax6* (Paired Box 6) in mouse ESCs or iPSCs led to the generation of retinal ganglion-like cells [15,16]. Parameswaran et al. used a two-step approach: they first induced iPSCs to generate RPCs in vitro, then acquired RGCs and photoreceptors by co-culturing the RPCs with mouse retinal explants [17]; however, these methods may bring potential risks to stem cell therapies due to the introduced exogenous genes (Figure 1B). Researchers started to seek other ways to generate retinal cells without changing the genome, i.e., using small molecules to induce cell differentiation.

Amirpour et al. treated hESCs with SHH (Sonic Hedgehog) to induce RPCs, and transplanted the RPC-derived cone cells into the rabbit eye to partly restore the visual function [18]. After treatments with DKK-1 (Dickkopf WNT Signaling Pathway Inhibitor 1), noggin, and DAPT (n-(n-(3,5-Difluorophenacetyl)-l-alanyl)-s-phenylglycine t-butyl Ester, a γ-secretase inhibitor), *Atoh7* (Atonal BHLH Transcription Factor 7)-misexpressing mouse iPSCs differentiated into RGCs. The RGCs were likely immature or dysfunctional. They were able to survive in the retina but rarely integrated into the neural network [19]. Besides DKK-1, noggin, and DAPT, other signaling and small molecules, such as Wnt (wingless-type MMTV integration site family), BMP (Bone Morphogenetic Protein), IGF (Insulin-Like Growth Factor), FGF (Fibroblast Growth Factor), RA (Retinoic Acid), and taurine, were used to generate photoreceptors or RPCs from iPSCs or hESCs [20,21,22,23,24] (Figure 1C). The induced photoreceptors could migrate, integrate, and form the layered functional cells within the host retina after transplantation, but the efficiency of integration was very low [25,26,27,28]. Apart from stem cells, RGCs [29] and photoreceptors [30] were also successfully derived from mouse embryonic fibroblasts and human fibroblasts in vitro.

Some researchers induced stem cells to form Embryoid Bodies (EBs) before acquiring RPCs and photoreceptors. For instance, it was found that inhibition of BMP and Wnt signaling in EBs led to the expression of eye field transcription factors, and later these cells spontaneously differentiated into retinal cells [31]. Osakada et al. applied small molecules to floating EBs to induce RPCs expressing typical cell markers RX (Retinal Homeobox Protein), MITF (Melanocyte Inducing Transcription Factor), PAX6, and VSX2 (Visual System Homeobox 2), and then finally specified the RPCs to photoreceptor fates with RA and taurine [32]. These protocols laid the foundation for the induction of 3-dimensional (3D) retinal organoids [33,34,35,36].

## 3. Methods to Induce and Optimize Retinal Organoids

### 3.1. The First Generation of Retinal Organoids

In 2011, Meyer et al. induced optic vesicle-like structures from hESCs. Progenitor cells in these structures expressed early-stage cell markers of retinal development, and could further differentiated into photoreceptor-like cells; however, many of these structures were forebrain-like and did not form the retina-like layers [35]. Sasai et al. successfully generated 3D optic cups from mouse ESCs [37,38]. Later, by a similar approach, they obtained optic cups derived from hESCs [2] (Figure 1D). Since then, researchers worldwide have invented different ways of inducing retinal organoids, which are nicely summarized in Llonch’s review for further reading [39].

### 3.2. Categories of Major Methods of Inducing Retinal Organoids

Currently, retinal organoid induction methods can be classified into three categories. The first category adapts a 2D to 3D process, but does not go through an EB stage [40,41]. In brief, after iPSCs are cultured to 70% confluence, the Essential 8 medium is replaced with the Essential 6 medium (without FGF2 and TGFβ). Two days later, the N2 supplement is added. By about 4 weeks, self-forming neuroepithelial-like structures appear in the culture dishes. These structures are picked up and cultured in suspension media containing B27 (3D process). It was reported by some groups that the organoids formed a rosette-like structure, and the layers were disordered, but the photoreceptors underwent a normal differentiation course [40,41]; however, the disordered structures possibly had nothing to do with the protocol itself, and instead were caused by the application of Notch inhibitor DAPT at an early stage, which was reported to disrupt retinal organoid lamination [42].

The second category includes an EB stage [43]. Briefly, the ESCs are dissociated into small clumps and cultured in an mTeSR1 medium containing Blebbistatin to form EBs. Then, the EBs are gradually transitioned into neural induction medium to form EB aggregates. On day 7, EBs are plated onto coated (e.g., Matrigel) flasks or dishes until horseshoe-dome-shaped NR domains are formed (in about 4 weeks). The retina-like structures are dissected and collected in suspension for long-term culture.

The third category is the classic procedure reported by Sasai et al. [2,44]. Briefly, ESCs are dissociated into single cells, and quickly re-aggregated in gfCDM + KSR medium in low-cell-adhesion V-bottomed 96-well plates. The addition of BMP4 during the early phase (day 6) leads to the high efficiency of neuroepithelium induction. Its concentration is diluted into half by half medium change every 3–4 days. On day 18, NR-like tissues are dissected with fine forceps and scissors under a stereo microscope. They are cultured for 6 days in an RPE-induction medium then in a retina maturation medium. At around day 30, RGCs can be detected, and around day 130 photoreceptor cells can be seen. Organoids produced by methods of the latter two categories have clearly layered structures resembling those of retinas developed in vivo.

Modification and optimization of these methods are actively ongoing. For instance, rather than dissociating the ESC/iPSC aggregates into single cells in Sasai’s protocol, Kim et al. replaced the step with an incomplete dissociation of iPSCs/hESCs aggregates, and the final organoids were cone-rich [45].

## 4. Retinal Organoids as Disease Models

### 4.1. Retinal Organoids Recapitulate In Vivo Retinal Development

One obvious and serious question that needs to be addressed is what are the similarities and differences at the tissue, cellular, molecular and functional levels between retinal organoids generated in vitro and retinas developed in vivo. Using bulk RNA-seq, Welby et al. showed that purified cone cells derived from human retinal organoids had a similar transcriptome with fetal cone cells [4]. In similar approaches, more studies agree that mouse and human retina organoids have comparable laminar structures, cell types, transcriptomes with retinas [46,47,48]. Sridhar et al. performed single-cell RNA sequencing (scRNA-seq) to compare the transcriptomes of human fetal retinas, hiPSC-derived retinal organoids, and long-term cultured retinas. They found that the proportions of the various cell types were similar in the organoids, fetal retinas, and cultured retinas of equivalent developmental stages, though the level of cell-type-specific gene expression showed some differences [49]. Kim et al. reported a cone-rich retinal organoid and the scRNA-seq results showed that cones from these organoids had similar transcriptomes to those of the human macula [45]. Different from Kim’s work, Eldred et al. established a protocol for the generation of cone-rich retinal organoids by enhancing thyroid hormone signaling, which mimics endogenous development of cone cells [50]. These studies demonstrated that cells from retinal organoids share a common transcriptome with endogenous retinal cells; however, comparison studies of their functions are relatively sparse.

Another question is whether retinal organoids could recapitulate in vivo retinal development. Cui et al. performed transcriptome and cluster analyses, and showed that the retinal organoids developed slower than the native retina. One possible reason for that might be the loss of local nutrients [51]. Xie et al. performed chromatin accessibility analyses of human retinas and hiPSC-derived retinal organoids, and showed that retinal organoids recapitulated the time courses of human retinas, including retinal morphogenesis, retinal neurogenesis, and photoreceptor differentiation, but with divergent chromatin features. Further, the transcription factors regulating retinal development were conserved, and transcriptional networks were highly correlated between the developing human retinas and retinal organoids [52]. Cowan et al. developed light-sensitive human retinal organoids and performed scRNA-seq. They demonstrated that cell types in organoids matured in vitro to a stable developmental state at a rate similar to human retina development in vivo [53].

Though slight differences existed in results from various laboratories due to cell lines, procedures, protocols, and experiment operators, there is an incredible consistency in data that shows that retinal organoids resemble retinas in many aspects and can well recapitulate in vivo retinal development.

### 4.2. Disease Modeling with Retinal Organoids

Retinal organoids can serve as an in vitro tool to examine new drugs and therapies before their clinical application. Researchers have already modeled some diseases, such as enhanced S-cone syndrome, retinitis pigmentosa, Leber congenital amaurosis, glaucoma, retinoblastoma, and X-linked juvenile retinoschisis. They often used patient-derived cells to generate organoids, and attempted to rescue the diseased features with different strategies.

#### 4.2.1. Enhanced S-Cone Syndrome

Enhanced S-cone syndrome is a disease with a reduction in rod photoreceptors, an over-expansion of the S-cone population, and a varying degree of disturbance in M- and L-cone development. One of the common causative mutation genes is transcription factor *NR2E3* (Nuclear Receptor Subfamily 2 Group E Member 3) that controls the terminal differentiation and maturation of rod cells [54,55]. Bohrer et al. developed a CRISPR (Clustered Regularly Interspaced Short Palindromic Repeats)-based homology-directed repair strategy and corrected the *NR2E3* premature mutation (c.119-2-A>C) in patient-derived iPSCs. The organoids derived from the gene-corrected iPSCs regained the ability to express wild-type *NR2E3* [56]. *NRL* (Neural Retina Leucine Zipper) mutations also cause enhanced S-cone syndrome and retinitis pigmentosa, as it is critical for rod fate determination and maintenance [57,58]. Kallman et al. utilized *NRL*-deficient iPSCs to generate retinal organoids, and these organoids developed S-opsin-dominant photoreceptor populations as in *NRL*-deficient retinas [59]. They further identified *MEF2C* (Myocyte Enhancer Factor 2C) as a candidate regulator of cone development by organoid disease models [59]. Patient- or mutant-cell-derived retinal organoids could successfully recapitulate the diseased features, suggesting that they could be a good model for studying pathophysiologic mechanisms.

#### 4.2.2. Retinitis Pigmentosa

Retinitis Pigmentosa (RP) is an irreversible hereditary retinopathy caused by deleterious mutations in *TRNT1* (TRNA Nucleotidyl Transferase 1)*, RPGR* (Retinitis Pigmentosa GTPase Regulator)*, USH2A* (Usher Syndrome Type IIA; Usherin)*, PDE6B* (Phosphodiesterase 6B)*, RP2* (retinitis pigmentosa 2 homolog), and other genes. Sharma et al. found that patient-specific *TRNT1*-mutant iPSCs and iPSC-derived retinal organoids exhibited a deficit in autophagy [60]. Deng et al. generated iPSCs from three RP patients with *RPGR* gene mutations, and then differentiated them into retinal pigment epithelial cells and retinal organoids. Significant defects in photoreceptors and shortened cilia were observed. After mutations were corrected with CRISPR/Cas9 gene editing, photoreceptor structures, electrophysiological properties, ciliopathy, and gene expression were all partly rescued [61]. In order to examine the pathology of non-syndromic RP, mostly caused by mutations in *USH2A,* Guo et al. reprogrammed iPSCs from a patient’s cells with a mutation in *USH2A* (c.8559-2A > G/c.9127_9129delTCC), and generated retinal organoids. The expression of laminin in the *USH2A* mutant organoids was significantly lower, and the retinal pigment epithelium (RPE) cells also showed an abnormal morphology [62]. Gao et al. generated retinal organoids from late-onset RP proband-derived iPSCs with a *PDE6B* mutation. They performed transcriptome analyses and revealed a remarkably distinct gene expression profile in the mutant. It is the first late-onset RP model established, providing new insights into the *PDE6B*-related mechanism of RP [63]. *RP2* mutations cause a severe form of X-linked retinitis pigmentosa whose pathogenesis is unclear. Lane et al. produced gene-edited isogenic *RP2* knockout iPSCs and derived organoids. Outer nuclear layer (ONL) thinning following a peak in rod photoreceptor cell death was observed in the mutant organoids, and could be rescued by adeno-associated virus (AAV)-mediated gene augmentation with human *RP2* [64]. The evidence from organoid modeling thus demonstrates that the pathogenesis of RP is very divergent in distinct gene mutations. Further, disease models using patient-cell-derived retinal organoids provide a platform for testing treatments, such as CRISPR/Cas9- or AAV-mediated gene editing. For instance, the G56R mutation in *NR2E3* is one of the most common mutations causing autosomal dominant RP. Diakatou et al. generated by CRISPR/Cas9 editing allele-specific knockout of the mutant *G56R* allele in patient iPSCs and found that this knockout did not affect the differentiation potential of retinal organoids or NR2E3 expression [65].

#### 4.2.3. Leber Congenital Amaurosis

Leber Congenital Amaurosis (LCA) is an eye disorder that primarily affects the retina. Patients have photophobia, involuntary movements of the eyes, extreme farsightedness, and other visual impairments beginning in infancy. Mutations in genes *CEP290* (Centrosomal Protein 290) and *AIPL1* (Aryl Hydrocarbon Receptor-interacting Protein-like 1) are common causes of LCA. To investigate disease mechanisms of LCA and evaluate candidate therapies, Parfitt et al. collected differentiated photoreceptors in retinal organoids and RPE derived from hiPSCs with a common *CEP290* mutation (c.2991 + 1665A > G). They found that the high levels of aberrant splicing and cilia defects could explain the pathogenesis and treatment with an antisense morpholino could efficiently restore the function of *CEP290* [66]. Later, Dulla et al. identified a splicing-modulating oligonucleotide that could reduce aberrant splicing, increase wild-type CEP290, and restore photoreceptor ciliogenesis in retinal organoids [67]. Shimada et al. modeled cilia dysfunctions caused by *CEP290* mutations (IVS26 + 1655A > G) using ciliopathy-patient-cell-derived retinal organoids. They found that CEP290 protein was reduced in LCA fibroblasts with no detectable impact on cilia; however, the patient’s retinal organoids displayed less developed photoreceptor cilia [68]. Mutations in *AIPL1* cause Leber congenital amaurosis type 4 (LCA4), and in order to recapitulate the disease phenotype in vitro, Lukovic et al. generated hiPSCs and retinal organoids from an LCA4 patient with a Cys89Arg mutation in *AIPL1*. Consistent with phenotypes from the *AIPL1* animal model, the mutant *AIPL1* resulted in a decrease in the PDE6 complex in the organoid [69]. These studies suggest that retinal organoids serve as a resource to investigate the molecular mechanism of disease and safely test novel therapies in vitro.

#### 4.2.4. Glaucoma

RGCs connect the eye to the brain, and this connection is disrupted in glaucoma. The E50K mutation in the *OPTN* (Optineurin) gene is a leading cause of inherited glaucoma. VanderWall et al. used the CRISPR/Cas9 gene-editing technique to introduce the OPTN-E50K mutation into existing lines of hiPSCs and generated isogenic controls from patient-derived lines. They then derived retinal organoids from these cells. Numerous neurodegenerative deficits, including neurite retraction, autophagy dysfunction, apoptosis, and increased excitability, were observed in RGCs differentiated from OPTN-E50K hiPSCs, showing the utility of OPTN-E50K RGCs as an in vitro model of neurodegeneration [70]. These data suggest that RGCs in retinal organoids can recapitulate the pathogenesis of inherited glaucoma.

#### 4.2.5. Retinoblastoma

Retinoblastoma (RB) is a rare form of eye cancer that begins in the retina and most commonly affects young children. It is caused by mutations in the *RB1* (Retinoblastoma 1) gene. To test new therapies, Saengwimol et al. established and characterized a retinal organoid model derived from chemotherapy-naïve tumors. These organoids had histological features, DNA copy-number alterations, gene and protein expression, and drug responses, all consistent with those of tumor cells [71]. To study the role of *RB1* in early human retinal development and tumor formation, Zheng et al. generated retinal organoids from CRISPR/Cas9-derived *RB1*-null hESCs. Even though widespread apoptosis and reduced numbers of photoreceptor, ganglion, and bipolar cells were observed, retinoblastoma formation did not occur in organoids, suggesting that *RB1* deletion alone is not sufficient for tumor development [72].

#### 4.2.6. X-Linked Juvenile Retinoschisis

X-linked juvenile retinoschisis (XLRS), a degenerative retinopathy, is linked to mutations in the *RS1* (Retinoschisin 1) gene. From patients with XLRS, Huang et al. generated hiPSC-derived retinal organoids, which successfully recapitulated key features of XLRS, including retinal splitting, defective retinoschisin production, outer-segment defects, abnormal paxillin turnover, and impaired ER-Golgi transportation. CRISPR/Cas9 correction of the C625T mutation of the *RS1* gene normalized some of the diseased features [73].

All in all, researchers have already modeled the diseases mentioned above using patient- or mutant-cell-derived retinal organoids, investigated related gene functions, and tested promising therapies; however, gene mutations may not be the only contributing factor to such diseases. In vivo cell communications, extracellular environment, and some other aspects should also be considered in order to successfully recapitulate diseased phenotypes with retinal organoids in vitro.

## 5. Transplanting Retinal Cells Derived from Retinal Organoids

### 5.1. Transplanting Retinal Organoid Cells Purified by Surface Proteins

Fluorescent genes are exogeneous to primates and most other mammals. Their encoded proteins are more or less toxic to host cells and are not encouraged in clinical applications. In comparison, cell surface proteins are encoded by endogenous genes and can be recognized by specific antibodies and used in affinity purification. For instance, CD73 (ecto-5′-nucleotidase, Cluster of Differentiation 73) is a cell surface marker for cone/rod common precursors and mature rods in mouse, and can be harnessed to purify cells for transplantation [74]. Other studies found that CD73 combined with another cell surface marker CD24 (Cluster of Differentiation 24) was suitable for the purification of rod precursors [75,76]. Based on these results, a panel of cell surface markers was carefully examined to isolate and purify human photoreceptor precursors from retinal organoids and fetal retinas [77]. It was shown that CD73 alone was not sufficient for isolating photoreceptor precursors, suggesting that CD73 might also label non-photoreceptors in human retinal organoids (Day 100 and Day 200); however, Crx^+^; Rcvrn^+^ (Recoverin) double-positive photoreceptor cells took up 60% ± 14% of the population negatively selected by markers SSEA-1 (stage-specific embryonic antigen-1; Fucosyltransferase 4, Fut4; CD15) and CD29 (integrin beta 1, Itgb1). Moreover, the percentage of Crx^+^; Rcvrn^+^ population did not significantly increase by triple markers CD73^+^; SSEA-1^−^; CD29^−^. In fetal retinas, rod cells could be marked by CD73^+^; SSEA-1^−^; CD29^−^, but this panel could not mark any photoreceptors in retinal organoids. It is claimed that, compared to the positive selection strategy, negative selection avoids antibody carry-over that usually does harm to the survival of transplanted cells. Additionally, the negative strategy is also more favorable for the development of good manufacturing practices (GMP) [77]. Although CD73 labeled cone-like photoreceptors in *Nrl^−/−^* mouse retinas, it failed to identify cone cells in human fetal retinas or organoids [3]. Welby et al. isolated and compared cone photoreceptors from human fetal retinas and human retinal organoids by a cell surface biomarker panel SSEA1^−^; CD26^+^ (Dipeptidyl peptidase-4, DPP4); CD133^+^ (Prominin 1, Prom1); CD147^+^ (Basigin, Bsg), which positively enriched fetal L/M-opsin cones and a stem cell-derived cone photoreceptor population [4].

Cell surface antigen c-Kit (KIT proto-oncogene receptor tyrosine kinase, Kit; CD117) is another widely used cell surface marker. It is a type III receptor tyrosine kinase expressed in several types of stem cells [78]. It is a promising candidate for the screening of human RPCs, as previous studies have demonstrated that it marks a population of RPCs in developing mouse and human retinas [79]. Using the c-Kit^+^; SSEA^−^ marker panel, Chen et al. isolated from human fetal eyes a population of RPC stem cells that was able to proliferate and differentiate into all kinds of retinal cells in vitro. They also differentiated into photoreceptors that established synaptic connections with host bipolar cells, and partly restored retinal function when injected into the sub-retinal space of the Royal College of Surgeons rats [78]. It was found that at early stages following transplantation, these transplanted stem/progenitor cells released growth factors such as BDNF (Brain-Derived Neurotrophic Factor) and NGF (Nerve Growth Factor) to improve cell survival and function of the remaining structure of the host retina [80]. Using the same marker panel (c-Kit^+^; SSEA^−^), Chen et al. eliminated tumorigenic embryonic cells and enriched for RPCs from hESC-derived retinal organoids. When subretinally transplanted into *Pde6b*^rd1^ mouse models, these RPCs improved vision and preserved retinal structures. They also suppressed the activation of microglia and the production of inflammatory mediators, providing the grafted cells with a healthier microenvironment [79].

In order to discover more cell surface markers for photoreceptors, researchers conducted transcriptome analyses of different retinal organoids. As Kcnv2 (Potassium Voltage-Gated Channel Modifier Subfamily V Member 2) is a good marker for mouse photoreceptors, it can also be used for humans. Slc6a17 (Solute Carrier Family 6 Member 17; NTT4), Slc40a1 (Solute Carrier Family 40 Member 1; Ferroportin1, FPN1; Dusg), and Kcnh2 (Potassium Voltage-Gated Channel Subfamily H Member 2) have a higher expression in human photoreceptors compared to their relatively stable expression in mice. Human cones could be well identified with RTN4RL1 (Reticulon 4 Receptor-Like 1), ST3GAL5 (ST3 Beta-Galactoside Alpha-2,3-Sialyltransferase 5), GNGT2 (G Protein Subunit Gamma Transducin 2), and EPHA10 (EPH Receptor A10), while human rods might be marked by GABRR2 (Gamma-Aminobutyric Acid Type A Receptor Subunit Rho2) and CNGB1 (Cyclic Nucleotide Gated Channel Beta 1), two highly expressed cell surface molecules in mouse rods [81].

As for RGCs, researchers found that in retinal organoids, CD184 (C-X-C Motif Chemokine Receptor 4, CXCR4) has a dynamic expression profile related to different cell stages: it is enhanced in RGC-competent RPCs, and high in post-mitotic RGC precursors. As RGCs mature, another cell surface marker, CD171 (L1 Cell Adhesion Molecule, L1cam), becomes detectable. The temporally distinct expression of CD184 and CD171 allows better identification, enrichment, and purification of RGCs from precursors to maturing neurons in retinal organoids [82].

Currently, culturing retinal organoids requires tedious labor over a long period. To reach the GMP standard, some researchers committed to optimizing the means of cell purification, mass production, and transport [41,83,84,85,86,87,88]. It was reported that the developmental process of human retinal organoids was accelerated when co-cultured with mouse RPE cells [89]. Other studies explored long-distance transportation and cryopreservation of retinal organoids [90,91]. It was found that the freeze–thaw cycle of purified CD73^+^ cells resulted in a high percentage of cell death, whereas cryopreservation of retinal organoids led to better survival [92].

Not only appropriate donor cells but successful migration are required for correct integration [93]. Subretinally transplanted cells need to migrate from the transplantation site through the outer limiting membrane (OLM) to the ONL. The integrity of the OLM and the degree of host retinal gliosis are two determining factors affecting the integration process. By disrupting the integrity of OLM, the integration efficacy can be improved, but the strategies of doing so are not ideal for clinical applications. Gliosis of Müller glial cells decreases retinal integration, but may somehow promote survival of transplanted cells and remaining cones or RGCs [94,95].

### 5.2. Retinal Sheet Transplantation

A retinal sheet is usually a segment or part of tissue dissected from a fetal retina or cultured optic cup and preserves the neural circuitries and normal lamination structures. Compared to transplantation of single-cell suspension, there are some advantages for retinal sheet graft, such as less immune activation, a higher survival rate, longer life spans, and more suitability for therapies of late-stage retinal degeneration [96].

#### 5.2.1. Transplantation of Retinal Sheets in Animal Models and Human

A series of experiments have been performed using fetal retinal sheets with or without RPE. One important observation is that transplants of retinal sheets appeared to develop better lamination in the subretinal space than in the epiretinal space during treatment [97]. Several groups found that the vision of *Pde6b*^rd1^ mice could be partly restored, as the transplanted retinal sheets reconstructed synaptic connections with host retinas [98,99,100,101]. Similar results were achieved when human fetal retinal sheets (11–15.5 weeks of gestation) were transplanted into rats. These grafts differentiated to various types of retinal cells, including photoreceptors and improved rat vision [102]. Shirai et al. collected hESC-derived retinal sheets and transplanted them to monkey and rat retinas. In both animals, several retinal cell types differentiated from transplanted retinal sheets were observed. The photoreceptor cells of grafts migrated to the outer nuclear layer of host retinas and the host–graft synaptic connections were established [103]. McLelland et al. investigated the long-term (54–300 days) effect of grafting the hESC-derived retinal sheets in retinal degenerative (RD) rats and found that the grafts continued to grow and differentiate in host retina and improved the vision as determined by superior colliculus electrophysiological recording. In agreement with previous results, the grafts exhibited circular lamination (rosettes) similar to those of some fetal sheet transplants [104]. To investigate if immunosuppressive drugs could improve grafting outcomes, Singh et al. transplanted hESC-derived retinal sheets along with prednisolone plus cyclosporine A into cat retinas, and found that the drug combination lowered the immune response and increased survival of the grafts [105].

Neural ‘retinas’ from the retinal organoids instead of retinal sheets were used in some transplanting experiments. Takahashi et al. improved the method of generating mouse retinal organoids. ‘Retinas’ dissected from retinal organoids were transplanted into *Pde6b*^rd1^ mice and the host–graft neuronal connections were confirmed by immunohistochemistry [106,107]. Immune-suppressants dexamethasone (DEX) may also improve the survival of retinal cells after epiretinal transplantation. Xian et al. seeded ‘retinas’ from organoids onto Poly(Lactic-co-Glycolic) Acid (PLGA) scaffolds and implanted them into rhesus monkey eyes, some with Chronic Ocular Hypertension (OHT) induction. The survival and differentiation of these ‘retinas’ in OHT eyes were successful only with the DEX treatment, indicating that DEX is likely a promising immunosuppressant to enhance the survival of epiretinal implants [108].

Grafting of retinal sheets has been tentatively performed in clinical trials. In 2002, five RP patients who had only light perception in both eyes, received fetal retinal sheet graft. After 6 months, these patients did not show immune rejection, but the vision was not improved [109]. In 2008, 10 patients (six with RP and four with AMD) received a similar surgery, and 7 patients showed improved vision [110]. These results show that retinal sheet transplantation has the potential for application in therapies.

#### 5.2.2. Enhancing the Transplantation Effect by Neurotrophic Factors

Neurotrophic factors may improve the survival of transplanted retinal sheets. BDNF and GDNF (Glial-Derived Neurotrophic Factor) are two well-known neurotrophic factors that improve neuronal survival. In one study [111], the RPC retinal sheets of E19 rat were coated with PLGA microspheres with or without BDNF, and transplanted into the subretinal space of RD rats. Compared to non-BDNF-treated transplanted rats (57%), relatively more BDNF-treated transplanted rats (80%) responded to a ′′low light′′ intensity in a confined superior colliculus area. Although BDNF coating improved the functional efficacy of RPC grafts, the mechanism of the BDNF effect still needs to be elucidated [111]. Another study compared the effect of two trophic factors, BDNF and GDNF. RD rats (age 4–6 weeks) received subretinal transplants of intact sheets of fetal retinas treated with BDNF or GDNF. It appeared that GDNF exerted greater overall restoration than BDNF, as GDNF treatment improved responses of rats with both laminated and rosetted (more disorganized) transplants [112].

#### 5.2.3. The Pros and Cons of Retinal Sheet Transplantation

Although there are some advantages of transplanting retinal sheets (fetal retina-derived or organoid-derived) compared to single-cell suspension, the biggest problem so far is that retinal sheet grafts form rosettes in the host retina. In the rosettes, photoreceptors are located in the inner layer, preventing them from building proper synaptic connections with the host. One possible reason is the large size of retinal sheets restricting the rotation and migration of the graft, while transplanted single cells can easily adjust themselves in the host retina, migrating and integrating into the right lamina [93]. Another shortcoming of the organoid-derived retinal sheet is the lack of RPE cells that are normally wrapping the outer segments of photoreceptors but are damaged and needed to be replenished in patients with retinal degeneration; however, in the hESC-derived retinal organoids obtained with current technology, RPE cells are located in the opposite end of the organoid assembly and could not provide the protection and outer segment recycling for the optic cup. The third problem of retinal organoid sheet transplantation is the potential tumorigenicity. The ESCs or iPSCs are not guaranteed to be thoroughly removed from retinal organoid sheets, while it is relatively easier to eliminate them from single-cell suspension by fluorescence-activated cell sorting (FACS).

## 6. The Challenges of Current Retinal Organoid Technologies and Possible Solutions

Retinal organoids have great potentials and will play an increasingly important role in functional studies of gene mutations and the treatment of genetic diseases. Even though different retinal-organoid-producing methods have been reported, some non-negligible obstacles may greatly affect their application in drug development and therapies. First, the culture process requires immense labor and a lengthy duration. Acquisition of a large scale of retinal organoids is costly and time-consuming. Second, the inner cell layers, especially the ganglion cell layer, gradually degenerate when the organoids are cultured for a long term. The possible reasons are malnutrition and lack of synaptic connections with the optic center. Third, the retinal organoid lacks extra-retinal structures that are essential for the normal function and survival of the NR in vivo, such as RPE and blood vessels. The last and most important is heterogeneity among different cell sources and derived organoids. Because of heterogeneity and variation, it is hard to establish a stable retinal organoid model to study the disease mechanism and establish a standard in the high throughput screening to evaluate the drug effectiveness. Next, we elaborate on them one by one.

### 6.1. Immense Labor and a Lengthy Duration to Culture Mature Organoids

Currently, retinal organoid pick-up from plates and subsequent operations are exclusively carried out manually. This process is terribly inefficient and time-consuming. In high-throughput drug screening experiments, tens of thousands of organoids are routinely required, and obviously, this is beyond individual human ability. Recently, a research group developed a technology to generate thousands of retinal organoids from iPSCs in a single well of a 6-well plate [53]. This approach saves some culture plates but not tedious operations in the following steps; however, the same approach was reported by Regent et al., with fewer organoids produced [113]. The reason for the discrepancy needs further investigation. Close to human embryologic timelines, it generally needs 4–6 months, even a year, to generate a relatively mature human retinal organoid. An automatic robotic instrument should be developed to relieve researchers from manual operations to some extent.

### 6.2. Gradual Degeneration of Inner Cell Layers during Long-Term Culture

**Figure 2:**
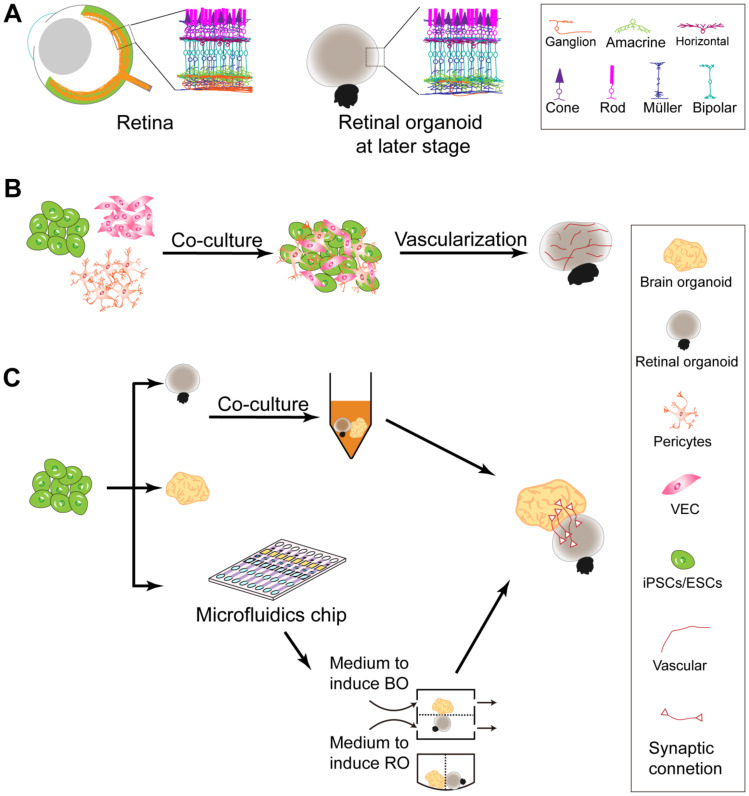
Strategies to retain the inner cell layers of retinal organoids. (**A**) Unlike native retinas, the inner cell layers of retinal organoids, which contain RGCs, amacrine cells, horizontal cells, and Müller cells, tend to degenerate during culture. (**B**) Reasons for the degeneration of inner cell layers include hypoxia and/or malnutrition. To solve the problem, iPSCs or ESCs may be reaggregated with vascular endothelial cells to form vascularized retinal organoids. More oxygen and nutrients diffuse into inner layers through blood-vessel-like tunnels and help the cells of the inner layers, especially RGCs, to survive longer. (**C**) Another reason for inner cell degeneration is the loss of synaptic connections to the brain. To establish the connections, brain and retinal organoids of the proper developmental stage may be co-cultured in a single 96-well. Alternatively, retinal and brain organoids are cultured in separate chambers but connected through channels in the microfluidic chip. Synaptic connections may be established to communicate retinal with brain organoids. BO, brain organoid; RO, retinal organoid; VEC, vascular endothelial cells.

One trouble of the current retinal organoid technique is the gradual loss of inner cells such as RGCs during long-term culture (Figure 2A). One possible reason is the lack of a vascular system during retinal organoid development, leading to hypoxia and malnutrition for the inner cells. Another reason might be the lack of sufficient synaptic connections between RGCs and the brain. The loss of RGCs in retinal organoids precludes the assessment of functional retinal circuit formation.

To solve the hypoxia and malnutrition problem, we propose that the retinal organoid could be vascularized by co-culturing with vascular endothelial cells and pericytes (Figure 2B). Vascularized brain organoids have been reported [114,115,116], in which oxygen and nutrition are transported into inner cell layers through vessel-like tunnels. To establish retina–brain organoid synaptic connections, the retinal and brain organoids can be co-cultured at the proper developmental stage, or in a microfluidics chip/chamber [117]. In the chip, the two organoids may be induced and cultured in separate but connected chambers with proper media. The synaptic connections could potentially be established during organoid development (Figure 2C). A similar study has been reported recently [118], which shows that RGC axons can extend into the thalamic region of retina–brain assembloids and that RGC survival is enhanced in such assembloids.

### 6.3. Lack of Interactions with Neighbor Tissues

The RPE plays several important roles in retinal homeostasis [119,120,121]. It functions as a retinal blood barrier, delivers blood-derived nutrients to photoreceptors, and transports ions, water, and metabolic products from the subretinal space to the blood. The RPE is also a source of growth factor release and phagocytosis of the outer segments of photoreceptors. A study of co-culturing RPE cells with retinal organoids has been reported [117]. In a microfluidic chip, RPE cells were plated under the retinal organoid, attaching to the photoreceptor out-segments. An active phagocytic uptake of outer photoreceptor segments was observed in this culture system. Except for the RPE, co-culture with lens organoid may also be helpful for the maturation of retinal organoid, which has not yet been attempted. Nevertheless, brain organoids containing bilateral optic vesicles have been reported recently. These organoids contain primitive corneal epithelial and lens-like cells, RPE, RPCs, axon-like projections, and electrically active neuronal networks [122]; therefore, they are expected to serve as valuable new tools for future studies of interactions between retinal organoids and their neighboring tissues.

### 6.4. The Varied Induction Efficiency and Cell Composition from Different Cell Sources

The induction efficiency is known to vary among different cell sources. It is speculated that the epigenetic background of iPSCs is a contributing factor to the varied results. Hiler et al. found that iPSCs derived from mouse rods were prone to differentiate into retinal organoids with fewer amacrine cells or cells of the inner nuclear layer, compared to mouse ESCs or fibroblast-derived iPSCs. Further results indicated that methylation played an important role in this process [123]. It was reported that the induction efficiency of five fibroblast-derived hiPSC lines (from different individuals) varied enormously, and the maturation of organoids was dependent on nutrition [124]. Li et al. observed that urine cell-derived hiPSCs were able to differentiate towards retinal fates and form 3D retinal organoids containing laminated NRs with all retinal cell types located in proper layers as in vivo, and generated highly mature photoreceptors with all subtypes [125]. With the use of a Notch inhibitor, DAPT, at an early timepoint and RA at a later stage, hiPSCs derived from keratinocytes with abnormal chromosomal content were permissive to the generation of 3D retinal organoids [126]. It was also demonstrated that Müller glial cell-derived iPSCs were able to differentiate toward the retinal fate and generate concomitantly both RPE and self-forming retinal organoid structures containing RPCs, with a modified retinal maturation protocol characterized by the presence of serum and high levels of glucose [127]. These studies show that the epigenetic background of iPSCs is a major factor contributing to the induction of retinal organoids, including the efficiency and cell composition. Aside from epigenetics, variation in organoid induction and differentiation may in part also be attributed to the heterogeneity of gene expression, since single hiPSCs were found to be heterogeneous in gene expression levels independent of their starting cell source, derivation technique, or passage number [128].

Optimization of medium supplements has been evaluated to minimize the variations. One is the introduction of Wnt signaling inhibitor DKK1 into the culture system, leading to a better induction and higher efficiency of some human iPSCs [129]. Dorgau et al. reported that culture media supplemented with decellularized retinal RPE and RPE-conditioned media significantly increased the generation of rod photoreceptors, while the addition of decellularized NR and RPE significantly enhanced ribbon synapse marker expression and the light responsiveness of retinal organoids, confirming that culture media may have a great impact on organoid development [130]. The usage of growth factors and the like has also been considered. Some researchers found that the addition of IGF1 (Insulin-Like Growth Factor 1) improved the induction efficiency [131]. Others found that organoids from three iPSC cell lines displayed a great variance, demonstrating that the differentiation to retinal organoids in response to IGF1 and BMP4 activation was line- and method-dependent [132].

These studies suggest that epigenetic background, gene expression heterogeneity, and signaling activities of iPSCs all play an important role in the success of retinal organoid induction. Although the addition of signaling molecules may be helpful to induce those obstinate iPSCs into retinal organoids, it is not a universally applicable strategy. One recommended strategy is to reprogram the primed hiPSC cells into a naive state that minimizes the epigenetic interference [133,134]. The current retinal organoid methods might be suitable for the naive state PSCs.

### 6.5. The Heterogeneity of Organoids in the Same Batch

Retinal organoids of the same batch may have considerable, sometimes tremendous, diversities in many aspects, including the sizes, the proportions of different cell types, and the developmental stages. As a result, for some gene mutations causing quantitative changes in features, it is confusing to figure out whether the phenotype is a result of the gene mutation, or just the variations between groups. It also brings more uncertainty to high-throughput drug screening. As mentioned previously, one common strategy to differentiate between variations and disease phenotypes is to generate isogenic controls by CRISPR/Cas9-mediated correction of gene mutation in patient-derived hiPSC lines. To solve the problem of variation in the developmental stage, a division of three developmental stages has been proposed based on morphology, gene expression, and culture time. Each has some typical characteristics that can be used to compare organoids of a similar developmental stage [135]; however, the definition of stages by itself is not precise or stringent, its application is limited in real practice.

**Figure 3:**
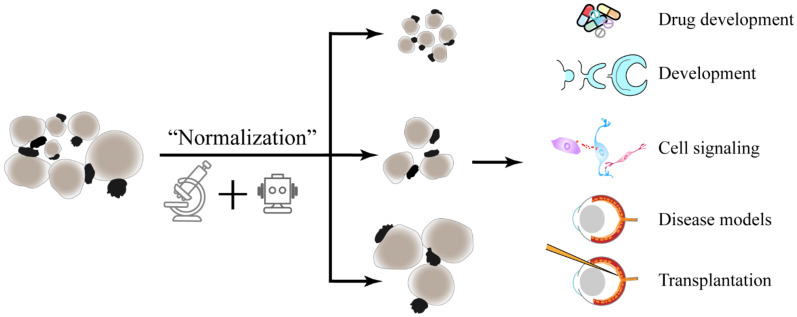
Normalizing heterogeneous retinal organoids by artificial intelligence. Heterogeneous retinal organoids are ‘normalized’ and grouped by their sizes, colors, fluorescent protein-tagged markers, or other features through image recognition and machine learning. These normalized organoids would decrease the variations during drug screening, disease models, developmental studies, and so on.

Aside from isogenic controls and marker gene expression, machine learning and image recognition may also be used to pick out organoids of similar size and developmental stage. Preliminary studies have been carried out by some groups [136,137,138,139]. These “normalized” retinal organoids can be applied to high-throughput compound screening and analyses of retinal diseases (Figure 3); however, to distinguish the causative result and variation associated with the phenotype, a golden rule of thumb is still that you have to increase the sample size to a sufficient amount.

## 7. The Next Generation Retinal Organoids

As discussed above, although the retinal organoid technology is moving forward rapidly, the main challenge remains to bridge the gap between the organoid with native retina. The next-generation retinal organoid would be expected to have a high level of structural and functional resemblance to retina and could faithfully substitute it for in vitro and in vivo studies with high reproducibility and replicability. Ideally, it would have an integrated vascular network, an increased 3D size if not close to native organ, a longer lifespan, and a pigment layer wrapping around.

To achieve the goal, it would be a shortcut to co-culture the PSCs or subsequent optic vesicles with vascular endothelial cells, RPE cells, or their precursors. The proportions of each cell type, the timepoints of co-culture, the bioactive media, and other influential factors should be systemically evaluated. A bigger size organoid demands a more dynamic environment for active exchanges of nutrients and metabolites. One solution is to use bioreactors or microfluidics that provide constant rolling of culture medium at a controllable speed [85,86]. Topographically structured scaffolds, controllable 3D matrices, organ-on-chip, bioprinting, and other measures should also be considered [140,141].

## 8. Summary

Retinal cells differentiated from stem cells in vitro are very useful in studies of developmental mechanism, disease pathogenesis, drug screening, and cell replacement therapies. Retrospectively, the development of retinal-cell-inducing technology can be divided into two stages by the landmark of retinal organoids. During the pre-organoid stage, the retinal progenitor cells or pluripotent stem cells are cultured traditionally and induced in a single layer or under a 2D condition. These cells differentiate into various types of retinal cells spontaneously. Or alternatively, specific retinal cell types are enriched under the guidance of misexpressed transcription factors and/or signaling molecules. The second stage is the introduction of the retinal organoid technique. The retinal organoids keep the laminated structures and recapitulate in vivo retinal development, which is particularly important for studying human retinal development and establishing retinal disease models under certain genetic backgrounds.

Although retinal organoid technology has made a great leap, there remain many problems to be solved, such as high heterogeneity between cell lines and experimental outcomes, the extensive culturing times, the progressive degeneration of inner cell layers, and the purification of target cells. We anticipate that, combined with microfluidics, vascularization, biomaterials, bioengineering, and other technologies, the next-generation retinal organoid technology will likely bridge the gap between organoids and retinas.

## Data Availability

Not applicable.

## Abbreviations

(h)ESCs	(human) embryonic stem cells
(h)iPSCs	(human) induced pluripotent stem cells
3D	three dimension(al)
AAV	adeno-associated virus
AIPL1	aryl hydrocarbon receptor-interacting protein-like 1
AMD	age-related macular degeneration
Atoh7	atonal BHLH transcription factor 7
BDNF	brain-derived neurotrophic factor
BMP	bone morphogenetic protein
CEP290	centrosomal protein 290
c-Kit	KIT proto-oncogene receptor tyrosine kinase
CNGB1	cyclic nucleotide-gated channel beta 1
CNGT2	G protein subunit gamma transducin 2
CRISPR	clustered regularly interspaced short palindromic repeats
Crx	cone-rod homeobox
Cxcr4	C-X-C motif chemokine receptor 4
DAPT	*N*-(*N*-(3,5-Difluorophenacetyl)-l-alanyl)-S-phenylglycine t-butyl Ester
DEX	dexamethasone
DKK-1	dickkopf WNT signaling pathway inhibitor 1
EBs	embryoid bodies
EPHA10	EPH receptor A10
FACS	fluorescence-activated cell sorting
FGF	fibroblast growth factor
GABRR2	gamma-aminobutyric acid type A receptor subunit rho2
GNDF	glial-derived neurotrophic factor
IGF	insulin-like growth factor
IGF1	insulin-like growth factor 1
INL	inner nuclear layer
Kcnh2	potassium voltage-gated channel subfamily H member 2
Kcnv2	potassium voltage-gated channel modifier subfamily V member 2
LCA	Leber congenital amaurosis
MEF2C	myocyte enhancer factor 2C
MITF	melanocyte inducing transcription factor
NR	neural retina
NR2E3	nuclear receptor subfamily 2 group E member 3
NRL	neural retina leucine zipper
OHT	ocular hypertension
OLM	outer limiting membrane
ONL	outer nuclear layer
OPTN	optineurin
Pax6	paired box 6
PDE6B	phosphodiesterase 6B
PLGA	poly(lactic-co-glycolic) acid
RA	retinoic acid
RB	retinoblastoma
RB1	retinoblastoma 1
Rcvrn	recoverin
RD	retinal degeneration(/tive)
RGCs	retinal ganglion cells
RP	retinitis pigmentosa
RP2	retinitis pigmentosa 2 homolog
RPCs	retinal progenitor cells
RPE	retinal pigment epithelium
RPGR	retinitis pigmentosa GTPase regulator
RS1	retinoschisin 1
RTN4RL1	reticulon 4 receptor Like 1
RX	retinal homeobox protein
scRNA-seq	single-cell RNA sequencing
SHH	sonic hedgehog
Slc40a1	solute carrier family 40 member 1
Slc6a17	solute carrier family 6 member 17
SSEA-1	stage-specific embryonic antigen-1
ST3GAL5	ST3 beta-galactoside alpha-2,3-sialyltransferase 5
TRNT1	TRNA nucleotidyl transferase 1
USH2A	Usher syndrome type IIA; Usherin
Vsx2	visual system homeobox 2
Wnt	wingless-type MMTV integration site family
XLRS	X-linked juvenile retinoschisis
